# 

**Published:** 1907-12-14

**Authors:** 


CONTENTS.

| | PAGE |
|---|---|
| **The True Position of the Specialist** | 277 |
| **St. George's Hospital** | 278 |
| **Annotations—** | |
| Glasgow University Club, London—Consultations at Hospitals —The Prevalence of Scarlet Fever | 279 |
| **Medical Opinion and Movement** | 280 |
| **Hospital Clinics—** | |
| Some Medical Hæmorrhages. By Leonard Williams, M.D., M.R.C.P. | 281 |
| **Points in Treatment—** | |
| Maragliano's Serum in Phthisis | 285 |
| **Clinical Points—** | |
| A Simple Method of Determining the Blood Coagulation time | 286 |
| **Points in Surgery—** | |
| The Treatment of Fractures from a Common-sense Point of View | 287 |
| Osteo-Arthritis | 288 |
| **Notes on Current Neurology—** | |
| "Rudimentary" Forms of Tabes | 289 |
| **Practical Notes on Diagnosis and Treatment** | 290 |
| **Diseases of Children—** | |
| Chronic Colitis | 291 |
| **Gynæcology—** | |
| Pernicious Vomiting of Pregnancy | 292 |
| **Otology—** | |
| Concerning Operation for Abscess of the Brain Associated with Otorrhœa | 293 |
| **Therapeutic Notes** | 294 |
| **The Royal Army Medical Corps Section—** | |
| The War Efficiency of the Medical Volunteer | 295 |
| **Resident Medical Officers' Department—** | |
| Infectious Diseases in General Hospitals | 296 |
| **Hospital Administration—** | |
| New Dental Hospital at Melbourne, Australia | 297 |
| Protection against Fire in London | 298 |
| The Incorporate Institute of Hygiene | 299 |
| News and Coming Events | 300 |
| The Graduates' Medical Schools | 300 |
| **Nursing Administration—** | |
| The Registration of Nursing Homes | 301 |
| **Editor's Letter-Box—** | |
| "Medical Education and the London University" | 302 |

